# Surrogate-Assisted Hybrid Meta-Heuristic Algorithm with an Add-Point Strategy for a Wireless Sensor Network

**DOI:** 10.3390/e25020317

**Published:** 2023-02-09

**Authors:** Jeng-Shyang Pan, Li-Gang Zhang, Shu-Chuan Chu, Chin-Shiuh Shieh, Junzo Watada

**Affiliations:** 1 College of Computer Science and Engineering, Shandong University of Science and Technology, Qingdao 266590, China; 2 Department of Information Management, Chaoyang University of Technology, Taichung 41349, Taiwan; 3 Department of Electronic Engineering, National Kaohsiung University of Science and Technology, Kaohsiung 80778, Taiwan; 4 Graduate School of Information, Production and Systems, Waseda University, Kitakyushu 808-0135, Japan

**Keywords:** gannet optimization algorithm, differential evolutionary algorithm, surrogate model, add-point strategy

## Abstract

Meta-heuristic algorithms are widely used in complex problems that cannot be solved by traditional computing methods due to their powerful optimization capabilities. However, for high-complexity problems, the fitness function evaluation may take hours or even days to complete. The surrogate-assisted meta-heuristic algorithm effectively solves this kind of long solution time for the fitness function. Therefore, this paper proposes an efficient surrogate-assisted hybrid meta-heuristic algorithm by combining the surrogate-assisted model with gannet optimization algorithm (GOA) and the differential evolution (DE) algorithm, abbreviated as SAGD. We explicitly propose a new add-point strategy based on information from historical surrogate models, using information from historical surrogate models to allow the selection of better candidates for the evaluation of true fitness values and the local radial basis function (RBF) surrogate to model the landscape of the objective function. The control strategy selects two efficient meta-heuristic algorithms to predict the training model samples and perform updates. A generation-based optimal restart strategy is also incorporated in SAGD to select suitable samples to restart the meta-heuristic algorithm. We tested the SAGD algorithm using seven commonly used benchmark functions and the wireless sensor network (WSN) coverage problem. The results show that the SAGD algorithm performs well in solving expensive optimization problems.

## 1. Introduction

WSNs are a product of the new era, combining elements of computing, communication, and sensors to provide monitoring functions. A WSN consists of many sensor nodes forming a multi-hop self-organizing network using wireless communication. The WSN collaboratively senses, collects, and processes information in the signal coverage area and sends it to an observer to achieve the monitoring of the target area. Compared to traditional networks, WSNs have the advantage of being easy to deploy and fault-tolerant. Users can quickly deploy a practical WSN with limited time and conditions. Once deployed successfully, WSNs do not require much human effort, and the network automatically integrates and transmits information. Therefore, WSNs are widely used in various environments to perform monitoring tasks such as searching, battlefield surveillance, and disaster relief through the transmission of signals.

In a WSN, coverage is the ratio of the signal coverage of sensor nodes to the detection area. Since the number of sensors and the signal radius of a single sensor are limited, researchers are very interested in maximizing the detection of the target area under the premise of a limited number of sensors [1]. Optimizing the location of sensor nodes can improve network coverage and save costs. Various meta-heuristic algorithms are now used to solve the sensor node deployment problem. For example, Zhao et al. [2] used a particle swarm optimization algorithm and variable domain chaos optimization algorithm to find the optimal locations of sensors to improve the coverage in a two-dimensional environment. As the research progresses, the deployment of 3D WSN nodes applicable to the real environment is getting more and more attention. However, as the two-dimensional problems become three-dimensional problems, increasing the solution’s dimensions increases the computational difficulty and computational time, so we need to seek more suitable methods to solve such problems.

The meta-heuristic algorithm does not depend on the structure and characteristics of the problem and has a powerful search capability in dealing with non-convex and nondifferentiable problems. It can give relatively optimal solutions for practical problems in a certain time and is therefore, widely used in many engineering problems, such as speed reducer design [3,4], cantilever beam design [5,6], and wireless sensor network design [7,8,9]. However, meta-heuristic algorithms to optimize various problems require the computation of fitness values, the time consumption of which usually increases significantly with the complexity of the problem, such as finite element analysis [10] and 3D WSN node deployment [11]. Trying to solve such problems is time-consuming and it often takes hours or even days to obtain a satisfactory result, which is a waste of resources and a challenge to the researcher’s patience. Therefore, to address this problem of long solution time for complex fitness functions, researchers have proposed a surrogate-assisted meta-heuristic algorithm to improve the utility of meta-heuristic algorithms for expensive problems [12].

Surrogate-assisted meta-heuristic algorithms have been developed rapidly in recent years, and they have been studied by many researchers and are widely used in real-world problems. The surrogate-assisted meta-heuristic algorithm fits the fitness function using a surrogate model (also called an approximation model or meta-model). Nowadays, the widely used surrogate models are the Gaussian process (GP) or Kriging process [13,14], the polynomial approximation model [15], the support vector regression model [16], the radial basis function (RBF) model [17,18], etc., along with hybrid surrogate models that combine some of the models mentioned above [19,20]. Many papers have evaluated various surrogate models [21,22]. As the dimensionality of the optimization problem increases, the RBF surrogate model can obtain a relatively good performance, and the subsequent contents of this paper involve the RBF surrogate model.

The surrogate-assisted meta-heuristic algorithm can be classified according to the use of all samples or local samples when constructing the surrogate model. That is, it can be one of three types: a global, local, or hybrid surrogate-assisted meta-heuristic algorithm. Yu et al. [23] proposed a global surrogate model combined with a social learning particle swarm optimization algorithm, restart strategies, and generation-based and individual-based model management methods into a coherently operating whole. Pan et al. [24] proposed an effective, local, surrogate-assisted hybrid meta-heuristic algorithm that finds the nearest neighbors of an individual from a database based on Euclidean distance and uses this local information to train the surrogate model, introducing a selection criterion based on the best and top set of information to choose suitable individuals for true fitness evaluation. Wang et al. [25] proposed a hybrid surrogate-assisted particle swarm optimization algorithm whose global model management strategy is inspired by active learning based on committees. The local surrogate model is built from the best samples. The local surrogate model is run when the global model does not improve the optimal solution, and the two models are alternated.

The process of generating samples that enable constructing a surrogate model is called infill criteria (sampling). The performance of the surrogate model depends heavily on the quality and quantity of the samples in the database, which can affect how well the surrogate model fits the true fitness function. As the sampled data points need to be evaluated by the true fitness before they can be added to the database, each act of adding points brings a high computational cost. Therefore, we must investigate the infill criteria to expect better samples to build the surrogate model. The sampling strategies are broadly classified into static and adaptive sampling (add-point). Static sampling means that the training points required to build the surrogate model are drawn simultaneously. Currently, the widely used static sampling methods include Latin hypercube sampling (LHS) [26,27], full factorial design [28], orthogonal arrays [29], central composite design [30], etc. Since the sample points and the constructed surrogate model are independent in static sampling, static sampling cannot obtain helpful information for unknown problems. Static sampling hopes that the samples can regularly cover the whole solution space. LHS is a hierarchical sampling method which can avoid the aggregation of sample points in a small area and will use fewer samples when the same threshold is reached while making the computation less complicated. We use LHS to initialize the surrogate model.

Infill criteria are the product of combining static sampling with an add-point strategy. Static sampling techniques first establish the initial surrogate model, and the samples are updated using a meta-heuristic algorithm guided by the current surrogate model. Then, the samples are sampled according to the add-point strategy, and the surrogate model is re-updated to improve the overall accuracy of the current surrogate model. Finally, the above process is repeated until the maximum number of true fitness evaluations is satisfied. As the add-point strategy uses the previous experimental results as a reference for subsequent sampling, adaptive sampling can construct a more accurate surrogate model using a small number of samples compared to the inexperience of static sampling. Modern scientists are interested in the study of add-point strategies. They have proposed the statistical lower bound strategy [31], the maximizing probability of improvement strategy [32,33], and the maximizing mean squared error strategy [34,35]. Based on the above discussion, this paper proposes a new adaptive sampling strategy based on the historical surrogate model information.

Several aspects, such as the choice of the surrogate model, the choice of the add-point strategy, and the choice of the meta-heuristic algorithm, determine the performance of the surrogate-assisted meta-heuristic algorithm. Among these, the meta-heuristic algorithm’s choice largely determines the surrogate-assisted algorithm’s performance. The surrogate-assisted meta-heuristic algorithm also performs differently when we choose different meta-heuristic algorithms. In recent years, researchers have proposed more and more meta-heuristic algorithms inspired by certain phenomena or natural laws in nature, from the classical GA [36,37], DE [38], and PSO [39,40,41] to the recently proposed GOA [42] and PPE [43]. PPE is a population evolution algorithm that imitates the evolution rules of phasmatodea populations, that is, the characteristics of convergent evolution, path dependence, population growth, and competition. These algorithms have shown powerful optimization capabilities. Using a single meta-heuristic algorithm to solve complex problems may follow a similar update strategy to update the samples and thus fall into a local optimum. Therefore, many researchers consider hybrid algorithms a research hotspot, such as combining the structures of two algorithms or connecting them in series. Zhang et al. [44] proposed a strategy to combine the WOA and SFLA algorithms, i.e., combining the powerful optimization capability of WOA with the interpopulation communication capability of SFLA, and the hybrid algorithm obtained a more powerful optimization capability than when the two algorithms were run separately. However, few hybrid algorithms have been applied to surrogate-assisted meta-heuristic algorithms to deal with costly problems. We combined the recently proposed GOA with the DE algorithm to design a surrogate-assisted meta-heuristic algorithm.

We propose a surrogate-assisted hybrid meta-heuristic algorithm based on the historical surrogate model add-point strategy, the main work for which was as follows.

1. An add-point strategy is proposed, in which the useful information in the historical surrogate model is retained and will be compared with the information in the latest surrogate model to select the appropriate sample points for true fitness evaluation.
2. Combining GOA and DE algorithms, we make full use of the powerful exploration ability of GOA and the exploitation ability of DE, and propose an escape-from-the-local-optimum control strategy to control the selection of the two algorithms.
3. Generation-based optimal restart strategies are incorporated, and some of the best sample information is used to construct local surrogate models.
4. The SAGD algorithm was tested in seven benchmark functions and compared with other surrogate-assisted and meta-heuristic algorithms, including statistical analysis and iterative curves. The SAGD algorithm was applied to the 3D WSN node deployment problem to improve the network signal coverage.

The rest of this paper is organized as follows. Section 2 introduces the RBF surrogate model, GOA, DE algorithm, and 3D WSN node deployment problem. Section 3 describes the proposed surrogate-assisted hybrid meta-heuristic algorithm based on the historical surrogate model add-point strategy. Section 4 compares the SAGD algorithm with other algorithms on seven benchmark functions and shows how it has been applied to the 3D WSN node deployment problem. Finally, conclusions are given in Section 5.

## 2. Related Techniques

### 2.1. RBF Surrogate Model

The radial basis function is a real-valued function that takes its value only in relation to the distance from the origin, which can be expressed as Φ(x)=Φ(∥x∥). If the origin here is some other special point c, the formula can be defined as Φ(x,c)=Φ(∥x−c∥), ‖. ‖ means the calculation of the distance, commonly used as the Euclidean distance. The common radial basis functions include Gaussian basis functions, linear basis functions, cubic basis functions, etc.

The RBF model performs well for nonlinear problems with small-scale data and scales well for large-scale problems [21]. Therefore, we use the RBF surrogate model to approximate the expensive objective function. The RBF surrogate model used in this paper is in the form of interpolation, defined as follows, given the data points $x_{1},x_{2},\ldots ,x_{n}\in R^{D}$ and their fitness values $f\left(x_{1}\right),f\left(x_{2}\right),\ldots ,f\left(x_{n}\right)$. The radial basis function can be fitted to the given data points and their fitness values using Equation (1).

(1) $$\hat{f}\left(x\right)=\sum_{i=1}^{n}\omega_{i}\varphi \left(∥x-x_{i}∥\right)+p\left(x\right)$$

where φ(.) and ‖.‖ denote the basis function and distance, respectively—the cubic basis function and Euclidean distance are used in this paper; and $\omega_{i}$ denotes the weight coefficient of the cubic basis function interpolation for the point $x_{i}$. p(x) is a linear polynomial that satisfies $\sum_{i=1}^{n}\omega_{i}p\left(x_{i}\right)=0$. The unknown term in the radial basis function interpolation formula can be found in Equation (2).

(2) $$\left(\begin{array}{cc} \Phi & P\\ P^{T} & 0 \end{array}\right)\left(\begin{array}{c} w\\ b \end{array}\right)=\left(\begin{array}{c} F\\ 0 \end{array}\right)$$

where Φ is a matrix with n rows and n columns, each element of the matrix $\Phi_{i,j}=\varphi \left(∥x_{i}-x_{j}∥\right),i,j=1,2,\ldots ,n$; $b={\left(b_{1},b_{2},\ldots ,b_{D+1}\right)}^{T}$ is the parameter vector of p(x) and $F={\left(f\left(x_{1}\right),f\left(x_{2}\right),\ldots ,f\left(x_{n}\right)\right)}^{T}$; P is the matrix of basis functions of p(x) at the interpolation points. An RBF model is obtained when the rank of P is D + 1.

### 2.2. Gannet Optimization Algorithm

The GOA is a new meta-heuristic algorithm inspired by the predatory behavior of gannet in nature. It mainly consists of two phases, exploration and exploitation. The exploration phase mathematizes the gannet’s U- and V-shaped diving strategies, the exploitation phase focuses on the gannet’s sudden rotation and random swimming characteristics, and the two stages are run alternately to search for the best area in the search space.

#### 2.2.1. Initialization Phase

The GOA starts from a set of randomly initialized solutions X and chooses whether to perform an exploration or exploitation phase according to probability. The GOA contains a memory matrix MX. When updates are performed using each update strategy, the new position found is not immediately updated for the current position. Still, the new position is compared with the fitness value of the current position, and the individual with the better fitness value is retained in the X matrix. The initialization phase assigns X to MX.

#### 2.2.2. Exploration Phase

When hunting, gannets often observe their prey in the air and choose where prey is dense to glide and rush into the water. After rushing into the water, gannets have two types of diving, namely, U-shaped dives and V-shaped dives. A U-shaped dive is a long and deep one, and a V-shaped dive has a short and shallow shape. Equation (3) is used here to represent the shape of the U-shaped dive of the gannet, and Equation (4) represents the shape of the V-shaped dive.

(3) $$a=2\times \cos\left(2\times \pi \times r_{1}\right)\times t$$

(4) $$b=2\times \;V\left(2\times \pi \times r_{2}\right)\times t$$

(5) $$t=1-\frac{It}{Tmax\_iter}$$

(6) $$V\left(x\right)=\left\{\begin{array}{c} -\frac{1}{\pi }\times x+1,x\in \left(0,\pi \right)\\ \frac{1}{\pi }\times x-1,x\in \left(\pi ,2\pi \right) \end{array}\right.$$

where cos is MATLAB’s cosine function, $r_{1}$ and $r_{2}$ are random numbers between 0 and 1, It is the current number of iterations, and Tmax_iter is the maximum number of iterations specified. As can be seen, *t* slowly decreases throughout the iterations until it reaches 0.

Once the two dive shapes are defined, the gannet updates its positions according to these two dive shapes. Since the gannet chooses both dive shapes with the same probability when feeding, a random number q is defined to indicate which dive shape is chosen.

(7) $$MX_{i}\left(t+1\right)=\left\{\begin{array}{cc} X_{i}\left(t\right)+u1+u2 & ,q\geq 0.5\\ X_{i}\left(t\right)+v1+v2 & ,q<0.5 \end{array}\right.$$

(8) $$u2=A\times \left(X_{i}\left(t\right)-X_{r}\left(t\right)\right)$$

(9) $$v2=B\times \left(X_{i}\left(t\right)-X_{m}\left(t\right)\right)$$

(10) $$A=(2\times r_{3}-1)\times a$$

(11) $$B=(2\times r_{4}-1)\times b$$

where u1 and v1 are random numbers between [−a,a] and [−b,b], respectively; $X_{i}\left(t\right)$ is the ith individual in the current population; $X_{r}\left(t\right)$ is a randomly selected individual in the current population; and $X_{m}\left(t\right)$ is the average position of individuals in the current population. $r_{3}$ and $r_{4}$ are random numbers between 0 and 1.

#### 2.2.3. Exploitation Phase

When a gannet hunts in the water, a flexible fish often performs a sudden turning action to escape from the pursuit. A parameter C is defined for the capture ability, which decreases with the number of iterations (see Equation (12)); cf indicates the centripetal force of the fish in the water.

(12) $$C=\frac{1}{cf\times t2}$$

(13) $$t2=1+\frac{It}{Tmax\_iter}$$

(14) $$cf=\frac{M\times vs.el^{2}}{L}$$

(15) $$L=0.2+\left(2-0.2\right)\times r_{5}$$

where M=2.5 kg is the common weight of the gannet in reality, vel=1.5m/s is the speed of the gannet diving in the water, and $r_{5}$ is a random number between 0 and 1.

Define a constant c. If the gannet’s capture capacity C is greater than or equal to c, then the algorithm executes the strategy of a sudden turn to update the current position of the gannet. Otherwise, it means that the gannet does not have enough energy to complete the pursuit of the cunning fish at this time, so it carries out the strategy of random wandering to update the position of the gannet—that is, Equation (16).

(16) $$MX_{i}\left(t+1\right)=\left\{\begin{array}{cc} t\times \;\mathrm{delta}\;\times \left(X_{i}\left(t\right)-X_{\mathrm{Best}\;}\left(t\right)\right)+X_{i}\left(t\right) & ,C\geq c\\ X_{\mathrm{Best}\;}\left(t\right)-\left(X_{i}\left(t\right)-X_{\mathrm{Best}\;}\left(t\right)\right)\times P\times t & ,C<c \end{array}\right.$$

(17) $$delta=C\times \left|X_{i}\left(t\right)-X_{\mathrm{Best}\;}\left(t\right)\right|$$

(18) P=Levy(Dim)

where $X_{\mathrm{Best}\;}\left(t\right)$ is the best individual in the current population, and Levy() is the Levy flight function used to model the behavior of individuals wandering randomly, which can be found in Equation (19).

(19) $$\mathrm{Levy}\left(\mathrm{Dim}\right)=0.01\times \frac{\mu \times \sigma }{{\left|v\right|}^{\frac{1}{\beta }}}$$

(20) $$\sigma ={\left(\frac{\Gamma \left(1+\beta \right)\times \sin\left(\frac{\pi \beta }{2}\right)}{\Gamma \left(\frac{1+\beta }{2}\right)\times \beta \times 2^{\left(\frac{\beta -1}{2}\right)}}\right)}^{\frac{1}{\beta }}$$

where $\mu ∼N\left(0,\sigma^{2}\right),vs.∼N\left(0,1\right)$, β=1.5 is a pre-defined constant, Γ is the gamma function that comes with MATLAB, and Dim is the dimensional size of the problem.

The GOA consists of two stages: exploration and exploitation, and each stage contain two update strategies. Algorithm 1 gives the pseudo-code of the GOA.
**Algorithm 1** Pseudo-code of the GOA.**Input:** 
N: population size; Dim: problem dimension; Tmax_iter: maximum number of iterations;**Output:** 
The position of the best individual and its fitness value;  1:Initialize the population X randomly, r and q are all random numbers from 0 to 1;  2:Generate memory matrix MX;  3:Calculate the fitness value of X;  4:**while** stopping condition is not met **do**  5:      **if** r>0.5 **then**  6:         **for** ${\mathrm{MX}}_{i}$ **do**  7:             **if** q≥0.5 **then**  8:                   Update the location Gannet using Equation (7),where q≥0.5;  9:             **else**10:                   Update the location Gannet using Equation (7),where q<0.5;11:             **end if**12:         **end for**13:    **else**14:         **for** ${\mathrm{MX}}_{i}$ **do**15:             **if** C≥0.2 **then**16:                   Update the location Gannet using Equation (16),where C≥0.2;17:             **else**18:                   Update the location Gannet using Equation (16),where C<0.2;19:             **end if**20:         **end for**21:    **end if**22:    **for** ${\mathrm{MX}}_{i}$ **do**23:          Calculate the fitness value of $MX_{i}$;24:          If the value of ${\mathrm{MX}}_{i}$ is better than the value of $X_{i}$, replace $X_{i}$ with ${\mathrm{MX}}_{i}$;25:    **end for**26:**end while**

### 2.3. Differential Evolutionary Algorithm

The DE algorithm was proposed by American scientist Rainer et al. to solve problems with Chebyshev polynomials, and it is widely used in shop scheduling, transportation, and engineering design problems. It has fast convergence, good robustness, and few control parameters. The DE algorithm has four basic steps: initialization, variation, crossover, and selection; and three important operations, variation, crossover, and selection, are performed cyclically. Mutation and crossover change the individuals, and the selection keeps the good individuals and eliminates the bad ones. The above operations lead the whole population to the optimal solution. These four steps are described in detail below.

The initialization of the population is the first step to be performed by the meta-heuristic algorithm. The DE algorithm is the same as most algorithms, in that it generates a population X by random initialization within predefined bounds. The second step is the generation of the mutation vector by the mutation operation. A mutation operation is an operation that selects several individuals and changes the basis vector given the difference between them. There are many kinds of mutation operations, and the common ones are shown in Equations (21)–(24).

(21) $$v_{i}^{t}=x_{\mathrm{rand}1\;}^{t}+f\cdot \left(x_{\mathrm{rand}2\;}^{t}-x_{\mathrm{rand}3\;}^{t}\right)$$

(22) $$v_{i}^{t}=x_{\mathrm{best}\;}^{t}+f\cdot \left(x_{\mathrm{rand}1\;}^{t}-x_{\mathrm{rand}2\;}^{t}\right)$$

(23) $$v_{i}^{t}=x_{\mathrm{rand}1\;}^{t}+f\cdot \left[\left(x_{\mathrm{rand}2\;}^{t}-x_{\mathrm{rand}3\;}^{t}\right)+\left(x_{\mathrm{rand}4\;}^{t}-x_{\mathrm{rand}5\;}^{t}\right)\right]$$

(24) $$v_{i}^{t}=x_{\mathrm{best}\;}^{t}+f\cdot \left[\left(x_{\mathrm{rand}1\;}^{t}-x_{\mathrm{rand}2\;}^{t}\right)+\left(x_{\mathrm{rand}3\;}^{t}-x_{\mathrm{rand}4\;}^{t}\right)\right]$$

where *f* is a scaling factor to control the scale of mutation; $x_{rand1}^{t},\ldots ,x_{\mathrm{rand}\;5}^{t}$ denotes the random individuals in generation t; rand1, rand2, rand3, rand4, and rand5 are different from each other; $x_{best}^{t}$ is the best individual in generation t. Thus, Equation (22) means that the best individual on the current population is the base vector, and two individuals are randomly selected to make the difference to form the difference vector, and the base vector is perturbed by the difference vector. This mutation operation is used later in this article.

The third step is the crossover operation, which uses the mutation vector generated in the second step to crossover with the target vector to generate the test vector. Two common crossover methods are binomial crossover and exponential crossover. The binomial crossover can be described as Equation (25).

(25) $$tr_{i,j}^{t}=\left\{\begin{array}{c} v_{i,j}^{t},\;\mathrm{if}\;\left(\;\mathrm{rand}{\;}_{i,j}\left(0,1\right)\leq CR\;\mathrm{or}\;j=j_{\mathrm{rand}\;}\right)\\ x_{i,j}^{t},\;\mathrm{otherwise}\; \end{array}\right.$$

where CR=0.9 is a predefined crossover probability; $rand_{i,j}\left(0,1\right)$ is a random number between 0 and 1. The above two variables jointly determine how many dimensions in the trial vector have information from the mutation vector. $j_{rand}$ is a random integer uniformly distributed in the interval [1,D], which is used to ensure that at least one dimension is updated in each crossover operation, and D represents a total of D dimensions.

The final selection operation uses the idea of greed by comparing the fitness values of the target vector and the test vector and selecting the one with the better fitness value for the next iteration. The pseudo-code of the DE algorithm is given in Algorithm 2.
**Algorithm 2** Pseudo-code of the DE.**Input:** 
N: population size; Dim: problem dimension; Tmax_iter: maximum number of iterations; F: scaling factor; CR: crossover probability;**Output:** 
The position of the best individual and its fitness value;  1:Initialize the population X randomly;  2:Calculate the fitness value of X;  3:**while** stopping condition is not met **do**  4:      **for** $x_{i}^{t}$ **do**  5:         The mutation vector $v_{i}^{t}$ is generated using the mutation operation shown in Equation (22) for individual $x_{i}^{t}$;  6:      **end for**  7:      **for** $x_{i}^{t}$ **do**  8:         A test vector ${\mathrm{tr}}_{i,j}^{t}$ is generated for individual $x_{i}^{t}$ and variance vector $v_{i}^{t}$ using the crossover operation shown in Equation (25);  9:      **end for**10:      Calculate the fitness value of the function for each trial vector ${\mathrm{tr}}_{i,j}^{t}$;11:      **for** $x_{i}^{t}$ **do**12:         Generate the next generation of individuals $x_{i}^{t+1}$ using the selection operation for individual $x_{i}^{t}$ and trial vector ${\mathrm{tr}}_{i,j}^{t}$;13:      **end for**14:**end while**

### 2.4. 3D Wireless Sensor Network Node Deployment

Wireless sensor networks are considered the second largest network after the Internet and one of the most influential technologies of the 21st century [45]. The optimization of WSNs includes routing and deployment optimization [46,47,48]. With a limited number of sensors, improving node coverage in WSNs has always been an important issue. Optimizing the coverage of WSNs is important for the rational allocation of network resources and better-accomplishing tasks such as environmental sensing and information acquisition. The research on 2D planar node deployment has achieved many desireable, and the application to 3D spatial coverage strategy has also gradually attracted the attention of scholars. Using meta-heuristic algorithms to study sensor deployment problems in 3D environments is a highly time-consuming study, so using surrogate-assisted meta-heuristic algorithms to solve such problems is a good direction.

**Figure 1:**
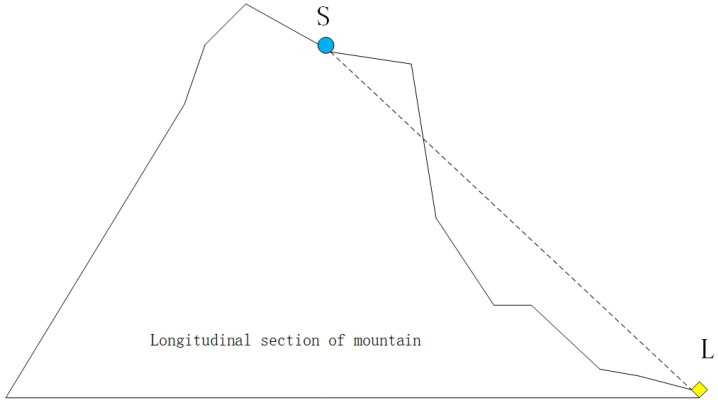
Three-dimensional terrain node signal obstruction example.

When a sensor detects the area around it, the emitted signal may be blocked by intermediate obstacles and thus unable to detect things within its coverage, so the signal coverage of the whole sensor network is affected. Here we use Bresenham’s line of sight (LOS) [11] algorithm to detect whether there is a spatial obstruction between two points. As shown in Figure 1, if there is a protruding obstacle between sensor node S and a point L within its communication radius, it blocks the detection of the point L by sensor node S. If no other sensor node detects this location either, then this location is a blind area for the whole sensor network. We use the 0-1 model to optimize the WSN coverage. In the 0-1 model, the sensing radius is set to $R_{s}$. If the Euclidean distance between sensor node S and detection point L is less than the radius $R_{s}$ and there is no obstruction in between, the probability of the model is one. In the inverse situation, the probability is 0. It can be expressed as Equation (26).

(26) $$F\left(S,L\right)=\left\{\begin{array}{cc} 1, & \left(D\left(S,L\right)<R_{S}\right)\;\mathrm{and}\;\mathrm{if}\;\mathrm{no}\;\mathrm{obstacle}\;\\ 0, & \;\mathrm{else}\; \end{array}\right.$$

where function *D* is used to calculate the Euclidean distance between *S* and *L*.

## 3. Proposed SAGD Method

**Figure 2:**
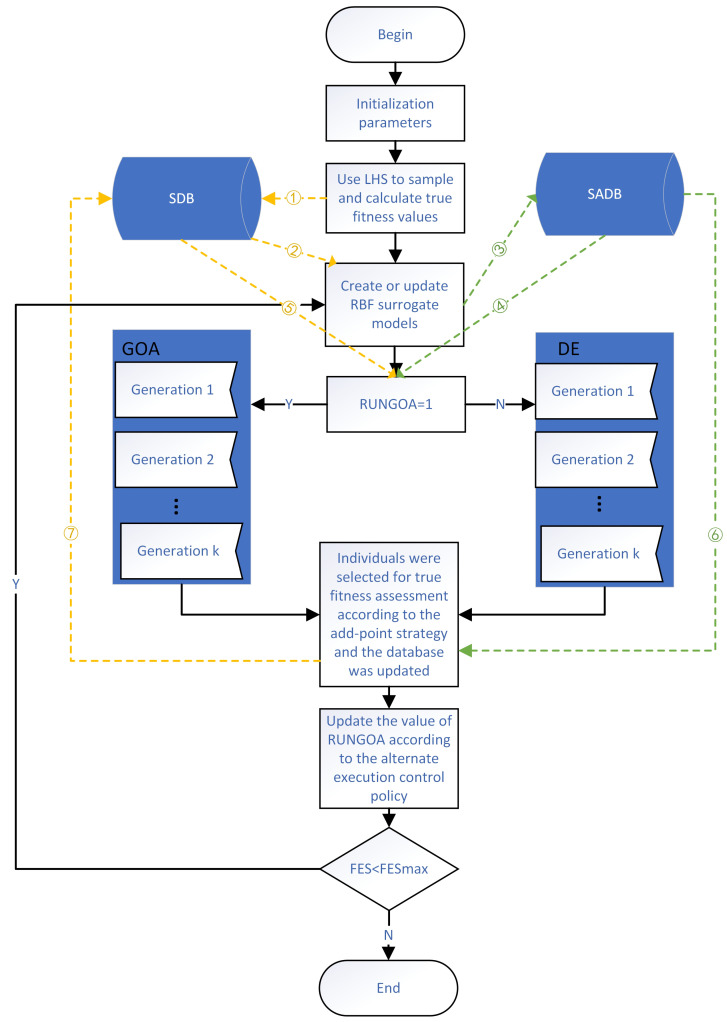
Framework of the algorithm SAGD.

This section introduces our proposed algorithm, which consists of four main parts: population initialization, surrogate model building or updating, execution of the hybrid meta-heuristic algorithm, and execution of the add-point strategy. The general framework of the algorithm SAGD is given in Figure 2, where the sample database (SDB) holds the data samples and their true fitness values, and the surrogate model database (SADB) keeps the information of all the surrogate models. The black line represents the algorithm stream, the green dashed line represents the surrogate model data stream, and the yellow dashed line represents the sample data stream.

First, in the population initialization phase, we initialize the solution space using the LHS method, where the LHS method uses the lhsdesign function that comes with MATLAB. The fitness values are then calculated for all individuals and the mean individual using the true fitness function. All individuals, the mean individual, and their fitness values are saved to the SDB, as shown in flow ➀ of Figure 2, for subsequent surrogate modeling and meta-heuristic algorithm initialization. The number of initial individuals is consistent with the population size of the meta-heuristic algorithm. The population size, ps, was set to 5 × D for the expensive optimization problems in 10, 20, and 30 dimensions. For the expensive optimization problems in 50 and 100 dimensions, the population size ps was set to 100+⌊D/10⌋.

The second step is the creation or updating of the surrogate model, where the data in the SDB are used for modeling, as shown in flow ➁ in Figure 2. The predictive power of the surrogate model is very important for the whole algorithm, so we want to build a good surrogate model with good performance. In general, the global surrogate model can fit the contour of the problem well, but it is difficult to apply the global surrogate model for high-dimensional problems, so we used local data to build the surrogate model in this study. The local surrogate model can speed up the search for promising regions and improve the accuracy of the surrogate model. When selecting the data for building the local surrogate model, the method of sample selection and the size of the data volume are both important. Here, we used the sample of neighbors of the current population in the SDB to build the local radial basis function surrogate model; the number of neighbors per individual was set to 5 × D for 10, 20, and 30 dimensions; and the number of neighbors was set to D for 50 and 100 dimensions, as shown in Algorithm 3. When updating the local surrogate model, we saved the information of the surrogate model at this time to the surrogate model database SADB and then updated the surrogate model. At the same time, the information was not saved when the surrogate model was built for the first time, as shown in flow ➂ in Figure 2.
**Algorithm 3** Pseudo-code for constructing a local radial basis function model.  1:**if** update the local surrogate model **then**  2:      The information of the local surrogate model at this time is kept for the add-point strategy;  3:**end if**  4:**for** each $x_{i}^{t}$ **do**  5:      Use Euclidean distance to find the n nearest neighbors of the individual from the database and form the set ${\mathrm{NP}}_{i}$;  6:**end for**  7:Merge the sets generated by each individual to form the training set NP;  8:Constructing a new local surrogate model with the training set NP;

The third step is to evolve the population using GOA and DE algorithms. The fitness function used here is the function fitted by the RBF surrogate model in the third step, as shown in flow ➃ in Figure 2. The GOA has a strong global search ability and better performance in high dimensions, and the position update model of the GOA can make its escape from a local optimum. Still, the GOA is more complicated. DE has good local searching and is fast. Still, it mostly perturbs the optimal individual, so the effect is not ideal when solving high-dimensional multimodal problems, and it will fall into a local optimum. Therefore, we combine the GOA and DE; i.e., we use the GOA or DE to evolve alternately. We propose a control strategy for alternate execution of the algorithms, where we start the DE algorithm if the locations of the individuals in the population updated by the GOA are present in the SDB and vice versa. We start the DE algorithm if the GOA does not find a better solution, and vice versa.

Here we add the optimal restart strategy based on generations after performing K generations. We select the individuals in the population at this time by the add-point strategy. We add the selected individual and the true fitness value to the SDB. At this time, the data in the database have changed. We select the best ps individuals from the database as the initialized population of the meta-heuristic algorithm to restart the evolution, as shown in flow ➄ in Figure 2. This generation-based optimal restart strategy prevents the GOA and DE from being misled by the errors generated by the fitted surrogate models. Using the best individual information in the SDB helps search for promising regions quickly. Again, because the local RBF model is updated every K generations, both meta-heuristic algorithms can fully explore the approximate fitness landscape.

As the evaluation of fitness is time-consuming in many complex problems, it is unacceptable for us to evaluate the true fitness value for unimportant individuals or individuals with no future, so it is important to select the appropriate individuals for evaluation. After K generations of meta-heuristic algorithm updates, our new add-point strategy for selecting appropriate individuals for true fitness evaluation kicks in. As the surrogate model is a fit to the real function, the historical surrogate model also contains useful information, and the predictive ability of the new surrogate model is usually better than that of the historical surrogate model. Thus, we use the information from the surrogate model database SADB for the sample selection, as shown in flow ➅ in Figure 2. If the fitness value obtained on the new surrogate model is better than that obtained on the historical surrogate model, the individual is proved to be more promising.

First, select the optimal individual in the population optimized by the GOA and DE algorithms, then randomly generate an integer tpc in the range of [1, ps] and calculate the average value of the first tpc individuals. The fitness values of the optimal individuals and the mean individuals are calculated using the historical surrogate model and the new surrogate model, respectively. Suppose the new surrogate model’s fitness value is better than the historical surrogate model’s. In that case, the fitness value of this individual on the true fitness function is calculated, and this individual and its true fitness value are saved in the SDB. Finally, suppose both the optimal individual and the mean individual are not added to the SDB. In that case, the fitness values of the new surrogate model for both individuals are inferior to the fitness values of the historical surrogate model. One individual is randomly selected among the first third of the entire population to calculate its true fitness value, and the random individual and its fitness value are saved to the SDB, as shown in flow ➆ in Figure 2. The pseudo-code of add-point strategy based on historical surrogate model is given in Algorithm 4.
**Algorithm 4** Add-point strategy based on historical surrogate model information.  1:Historical surrogate model information has been saved in SADB  2:Obtain the best individual in the current population (best), the mean of the top tpc individuals (mean), and the random individual in the top third (rand).  3:The fitness values of the optimal individual, the mean individual and the random individual were calculated using the new RBF surrogate model RBF_NEW and the historical RBF surrogate model RBF_OLD, respectively.  4:**if** RBF_NEW(best)<RBF_OLD(best) **then**  5:      Then the best individual and its true fitness value are saved to the SDB.  6:**end if**  7:**if** RBF_NEW(mean)<RBF_OLD(mean) **then**  8:      Save the mean individual and its true fitness value to SDB.  9:**end if**10:**if** Both the best individual and the mean individual were not added to the SDB **then**11:    Then the rand individual and its true fitness values are saved to the SDB.12:**end if**

It is worth noting that one or two sample points are added at a time in this add-point strategy. In the above steps, population initialization and surrogate model building are run only once. Then, surrogate model updating, hybrid meta-heuristic algorithm optimization, and the add-point strategy are executed iteratively. The process stops when the actual number of function calculations reaches the specified maximum number. We define that FESmax=11×D when D≤30, and FESmax=1000 when 50≤D≤100. Algorithm 5 gives the pseudo-code of the proposed SAGD algorithm.
**Algorithm 5** Pseudo-code for the SAGD algorithm.**Input:** 
RunGOA = 1; FES = ps + 1; gen = 0; K = 30; r: random values between 0 and 1;**Output:** 
Optimal solution and its fitness value;  1:The solution space is initialized by Latin hypercube sampling;  2:Use the real fitness function to calculate the fitness value for all individuals and the average individual;  3:**while**FES<FESmax**do**  4:      Initialize the population with the first ps samples in the SDB;  5:      **if** FES>ps+1 **then**  6:          Preserving the information of the local surrogate model and constructing the local surrogate model using Algorithm 3;  7:      **else**  8:          Putting populations and mean individuals and their true fitness values into a database;  9:      **end if**10:      **while** gen<K **do**11:         **if** RunGOA=1 **then**12:             **for** $X_{i}$ **do**13:                   **if** r>0.5 **then**14:                      Execution of Equation (7);15:                   **else**16:                      Execution of Equation (16);17:                   **end if**18:                   A local RBF surrogate model is used to estimate the fitness values of the evolved individuals and the original individuals, and the individuals with high fitness values are retained;19:             **end for**20:             gen = gen + 1;21:          **else**22:                **for** $X_{i}$ **do**23:                   Generation of a mutated individual by Equation (22);24:                   Generation of test individuals by Equation (25);25:                   A local RBF surrogate model is used to estimate the fitness values of the evolved individuals and the original individuals, and the individuals with high fitness values are retained;26:               **end for**27:               gen = gen + 1;28:         **end if**29:      **end while**30:      **if** When the meta-heuristic algorithm updates the location of the population individuals that are all present in the SDB **then**31:         then invert the value of RunGOA;32:      **else**  Calculating the true fitness value by selecting individuals using the add-point strategy of Algorithm 4 to update the SDB;33:         **if** The meta-heuristic algorithm did not find a better solution **then**34:             Invert the value of RunGOA;35:         **else**36:             Retain the value of RunGOA;37:         **end if**38:    **end if**39:**end while**

## 4. Experimental Results and Analysis

### 4.1. Benchmark Test Functions and Comparison Algorithms

In this section, we measure our algorithm using seven commonly used benchmark functions with different properties, the details of which are given in Table 1. These have been widely used to test the effectiveness of surrogate-assisted meta-heuristic algorithms. We followed the comparison method of the literature [23] in 10, 20, and 30 dimensions using the first five benchmark functions and in 50 and 100 dimensions for all benchmark functions except for Rastrigin. These functions are all minimization problems, and comparing the minima can help us evaluate the optimization algorithm’s effectiveness.

We compare the SAGD algorithm with the surrogate-assisted meta-heuristic algorithms that have been proposed and the ordinary meta-heuristic algorithms, where the surrogate-assisted meta-heuristic algorithms include GORS-SSLPSO [23] and FHSAPPSO [49], and the ordinary meta-heuristic algorithms include the GOA [42] algorithm and the DE [38] algorithm, which make up the SAGD algorithm. Among them, the GORS-SSLPSO algorithm uses generation-based evolutionary control and optimal restart strategy and combines social learning particle swarm for optimization; the FHSAPPSO algorithm uses a three-layer surrogate model, which is fuzzy surrogate-assisted, local surrogate-assisted, and global surrogate-assisted, and combines probabilistic particle swarm for optimization. Note that the GORS-SSLPSO and DE algorithms can be downloaded online, and the original authors provide the codes of FHSAPPSO and GOA.

All experiments were conducted on a computer with a 3.60 GHz Intel(R) Core(TM) i3-8100 CPU and 24.0 GB of RAM in a hardware environment and WIN10 operating system and MATLAB2021a in a software environment. For a fair comparison of the algorithms, we ran them 20 times and averaged them for comparison. We limited the number of iterations and the initial population size of all algorithms. The maximum number of iterations was 11 × D for dimensions 10, 20 and 30, and 1000 for dimensions 50 and 100. The population size ps was set to 5 × D for expensive optimization problems in 10, 20, and 30, dimensions; and 100+⌊D/10⌋ for expensive optimization problems in 50 and 100 dimensions. We set the number of generations, K, to the same value as in the literature [23], i.e., K = 30. Other setup parameters for the GORS-SSLPSO, FHSAPPSO, GOA, and DE were used as recommended in the original paper.

**Table 1:** Characteristics of seven benchmark functions.

Description	Dimension	Global Optimum	Characteristics
(Ellipsoid)$f\left(x\right)=\sum_{i=1}^{n}\left[x_{i}^{2}-10\cos\left(2\pi x_{i}\right)+10\right]$	10, 20, 30, 50, 100	0	Uni-modal
(Rosenbrock)$f\left(x\right)=\sum_{i=1}^{n-1}\left[100{\left(x_{i+1}-x_{i}^{2}\right)}^{2}+{\left(x_{i}-1\right)}^{2}\right]$	10, 20, 30, 50, 100	0	Multimodal with narrow valley
(Ackley)$f\left(x\right)=-20\exp\left(-0.2\sqrt{\frac{1}{n}\sum_{i=1}^{n}x_{i}^{2}}\right)-\exp\left(\frac{1}{n}\sum_{i=1}^{n}\cos\left(2\pi x_{i}\right)\right)+20+e$	10, 20, 30, 50, 100	0	Multimodal
(Griewank)$f\left(x\right)=\frac{1}{4000}\sum_{i=1}^{n}x_{i}^{2}-\prod_{i=1}^{n}\cos\left(\frac{x_{i}}{\sqrt{i}}\right)+1$	10, 20, 30, 50, 100	0	Multimodal
(Rastrigin)$f\left(x\right)=\sum_{i=1}^{n}\left[x_{i}^{2}-10\cos\left(2\pi x_{i}\right)+10\right]$	10, 20, 30, 50, 100	0	Multimodal
Shifted Rotated Rastrigin (SRR, Cost function see F10 in [50])	10, 20, 30, 50, 100	−330	Very complicated multimodal
Rotated Hybrid composition function with a			
Narrow Basin for the Global Optimum (RHC, Cost function see F19 in [50])	10, 20, 30, 50, 100	10	Very complicated multimodal

### 4.2. Experimental Results on 10D, 20D, and 30D Benchmark Functions

The experimental statistical results of the SAGD algorithm and the comparison algorithms GOA, DE, GORS-SSLPSO, and FHSAPPSO in 10, 20, and 30 dimensions, including the mean, best, worst, and standard deviation values obtained from multiple runs of each algorithm, are given in Table 2, Table 3 and Table 4. To statistically validate the results obtained by SAGD, we used the Wilcoxon rank sum test to discern whether the SAGD algorithm is significantly different from the other algorithms. The Wilcoxon rank sum test was performed at a significance level of alpha equal to 0.05. Using the symbol “+” indicates that SAGD is statistically significantly different from and better than the comparison algorithm. “=” indicates that SAGD is not statistically significantly different from the comparison algorithm. “−” indicates that SAGD is statistically significantly different from and inferior to the comparison algorithm. The Wilcoxon rank sum test results are also given in Table 2, Table 3 and Table 4.

Table 2, Table 3 and Table 4 show that the SAGD algorithm achieved promising results on all five benchmark functions relative to the other comparison algorithms. Specifically, the SAGD algorithm outperformed the other comparison algorithms on all 15 test problems. The SAGD algorithm not only achieved better average fitness values but also showed good stability by comparing standard deviations. This is because the SAGD algorithm combines two meta-heuristic algorithms to balance exploration and exploitation. The add-point strategy based on the historical surrogate model selected better sample points for the true fitness value calculation. For the common meta-heuristic algorithm, the surrogate-model-based meta-heuristic algorithm achieved better results on all test problems due to the inclusion of the surrogate model. The surrogate model can perform model estimation based on the evaluation of the true function and can better approximate the solution of the real function.

**Figure 3:**
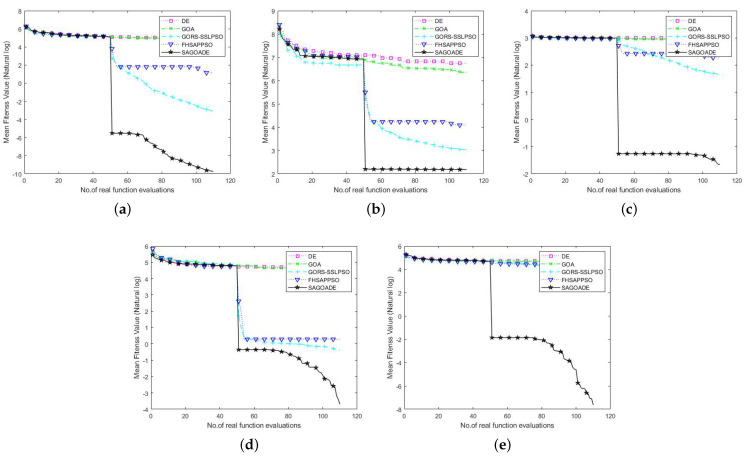
Convergence curves of SAGD, GOA, DE, GORS-SSLPSO, and FHSAPPSO for 10D benchmark functions. (**a**) Ellipsoid. (**b**) Rosenbrock. (**c**) Ackley. (**d**) Griewank. (**e**) Rastrigin.

**Figure 4:**
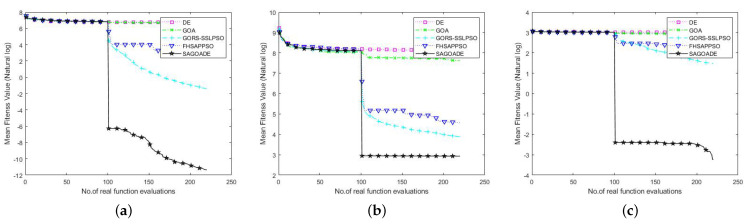

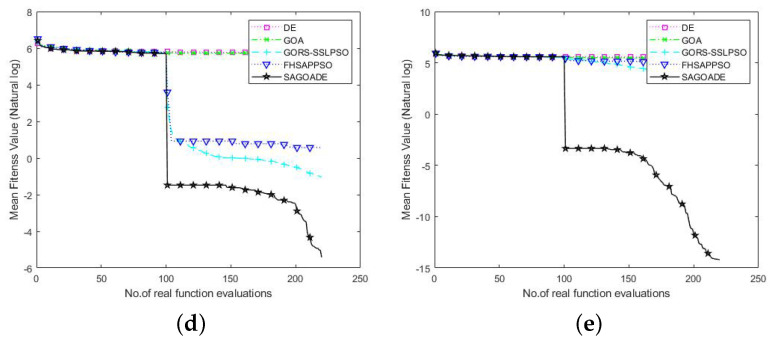
Convergence curves of SAGD, GOA, DE, GORS-SSLPSO, and FHSAPPSO for 20D benchmark functions. (**a**) Ellipsoid. (**b**) Rosenbrock. (**c**) Ackley. (**d**) Griewank. (**e**) Rastrigin.

**Figure 5:**
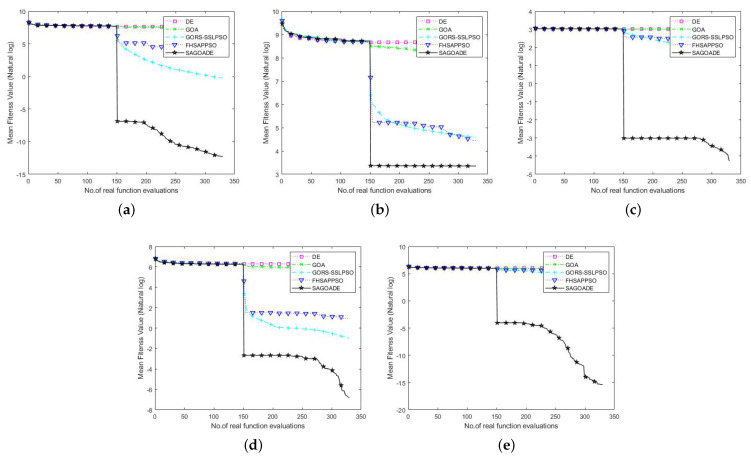
Convergence curves of SAGD, GOA, DE, GORS-SSLPSO, and FHSAPPSO for 30D benchmark functions. (**a**) Ellipsoid. (**b**) Rosenbrock. (**c**) Ackley. (**d**) Griewank. (**e**) Rastrigin.

To compare SAGD with the other algorithms more intuitively, we used the iteration curves of the average fitness values of the algorithms for a visual comparison. We can compare each algorithm’s convergence speed and ability on different benchmark functions through the convergence curve plots, as shown in Figure 3, Figure 4 and Figure 5. The horizontal axis is the number of iterations, and the vertical axis is the natural logarithm of the average fitness value.

In Figure 3, Figure 4 and Figure 5, we can see that our proposed algorithm has better convergence speed and convergence ability than other algorithms. The surrogate-assisted meta-heuristic algorithms all achieved relatively better solutions than ordinary meta-heuristic algorithms. Our algorithm had promising results on most of the tested problems, showing its superiority. The GORS-SSLPSO algorithm achieved better performance than the FHSAPPSO algorithm in most cases. With the information from the algorithm iteration comparison graphs, we can say that SAGD effectively solves low and medium-dimensional expensive optimization problems.

### 4.3. Experimental Results on 50D and 100D Benchmark Functions

To further test the scalability of the SAGD algorithm in higher dimensions, we compared the SAGD algorithm with GOA, DE, GORS-SSLPSO, and FHSAPPSO in two dimensions, 50D and 100D. The mean, best, worst, and standard deviation are given in Table 5 and Table 6, in regard to results for using a significance level alpha of 0.05 in Wilcoxon’s rank sum test. In Table 5 and Table 6, SRR is an abbreviated form of the shifted rotated rastrigin function, and RHC is an abbreviated form of the rotated hybrid composition function.

From Table 5 and Table 6, we can conclude that although the mean value of the GORS-SSLPSO algorithm on the 50 dimensions of the SSR benchmark function is not as good as that of the SAGD algorithm, it obtained a better standard deviation. The GORS-SSLPSO algorithm achieved better results than the SAGD algorithm on the 100 dimensions of the Rosenbrock and SSR benchmark functions. The SAGD algorithm has achieved good average fitness values on other test problems and has strong stability. Surrogate meta-heuristic algorithms also achieved more competitive results than GOA and DE. Our algorithm achieved no statistically significant difference in the 50 dimensions of the Rosenbrock function compared with GORS-SSLPSO. Our algorithm generated no statistically significant difference in the 100 dimensions of the SRR function compared with FHSAPPSO.

**Figure 6:**
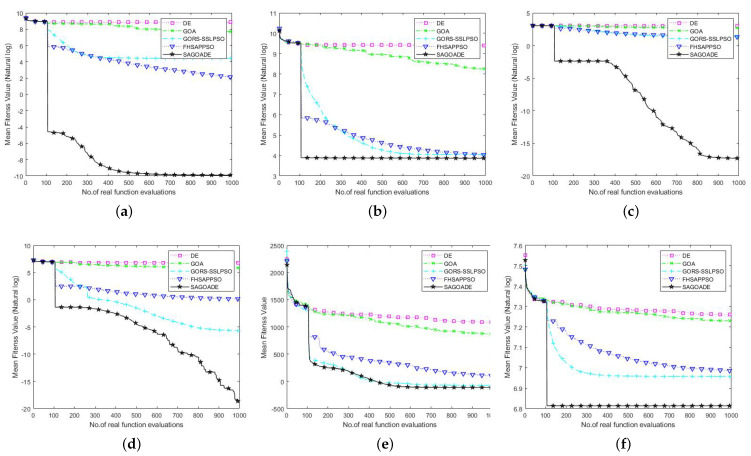
Convergence curves of SAGD, GOA, DE, GORS-SSLPSO, and FHSAPPSO for 50D benchmark functions. (**a**) Ellipsoid. (**b**) Rosenbrock. (**c**) Ackley. (**d**) Griewank. (**e**) SRR. (**f**) RHC.

**Figure 7:**
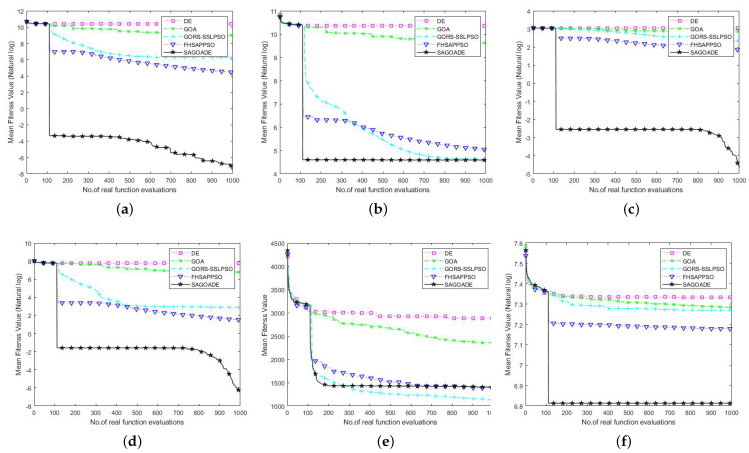
Convergence curves of SAGD, GOA, DE, GORS-SSLPSO, and FHSAPPSO for 100D benchmark functions. (**a**) Ellipsoid. (**b**) Rosenbrock. (**c**) Ackley. (**d**) Griewank. (**e**) SRR. (**f**) RHC.

The comparison graphs of the average fitness value iteration curves are given in Figure 6 and Figure 7. The horizontal axis is the number of iterations, and the vertical axis represents the natural logarithm of the average fitness value. The vertical axis of the SRR function represents the average fitness value. From Figure 6 and Figure 7, it can be seen that the SAGD algorithm has achieved a huge advantage over ellipsoid, Ackley, Griewank, and RHC benchmark functions, which shows a strong ability to explore and escape local optima. For the Rosenbrock and SRR benchmark functions, there was no significant gap between the proposed algorithm and the GORS-SSLPSO and FHSAPPSO algorithms in the later iteration curves. The SAGD algorithm won on the 50 dimensions of the Rosenbrock and SRR benchmark functions, and the GORS-SSLPSO algorithm won with 100 dimensions.

The above analysis shows that the SAGD algorithm is equally effective in high-dimensional expensive problems. In conclusion, the SAGD algorithm provides good solutions to low, medium, and high-dimensional expensive optimization problems. There are several reasons why our algorithm can provide better solutions. The first is that the combination of GOA and DE algorithms enhances the local exploitation ability and global search ability of the surrogate model; the control strategy of escaping the local optima subtly controls the alternation of GOA and DE. Secondly, the add-point strategy based on the database of the surrogate model can again select the appropriate samples for true evaluation. Finally, the generation-based restart strategy makes the surrogate model better use the information in the sample database.

### 4.4. Effects of the Hybrid of GOA and DE

**Figure 8:**
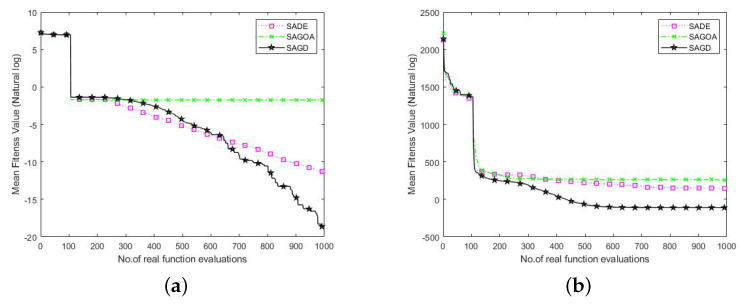
Convergence curves of SADE, SAGOA, and SAGD over 50D Griewank and SRR functions. (**a**) Griewank. (**b**) SRR.

**Figure 9:**
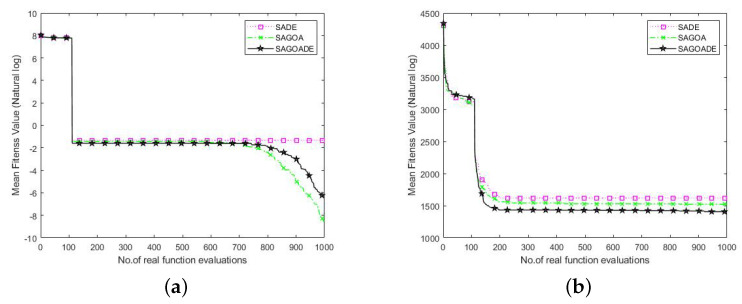
Convergence curves of SADE, SAGOA, and SAGD over 100D Griewank and SRR functions. (**a**) Griewank. (**b**) SRR.

To verify the validity of GOA and DE algorithms for SAGD, we performed validity analysis on 50 and 100 dimensions for both Griewank and SRR functions. The three algorithms compared were SADE, SAGOA, and SAGD. SADE the SAGD without the GOA, and SAGOA the SAGD without the DE algorithm. The average convergence curves obtained for these three algorithms in 20 independent runs are shown in Figure 8 and Figure 9. In the 50-dimension Griewank function plot, we can see that the three algorithms converged with similar performance in the early iterations. Still, our proposed algorithm showed strong competitiveness in the late iterations because the SAGD algorithm combines the unique advantages of the two algorithms and can balance exploration and exploitation well.

In the Griewank function plot in 100 dimensions, we can see that SAGOA outperformed the SAGD algorithm, which is due to the low exploration ability of the DE algorithm in higher dimensions, and the operation of the DE algorithm in the SAGD algorithm limits the number of GOA runs. The SRR plots for 50 and 100 dimensions show smoother declines of all three curves, but the SAGD algorithm outperformed the other two algorithms. The SADE was better than the SAGOA in lower dimensions, and the SAGOA was better than the SADE algorithm in higher dimensions. The above analysis shows that the combination of GOA and DE algorithms can compensate for the disadvantages of individual algorithms and improve the algorithm’s performance in most cases.

### 4.5. Application on Node Deployment in a Wireless Sensor Network

**Figure 10:**
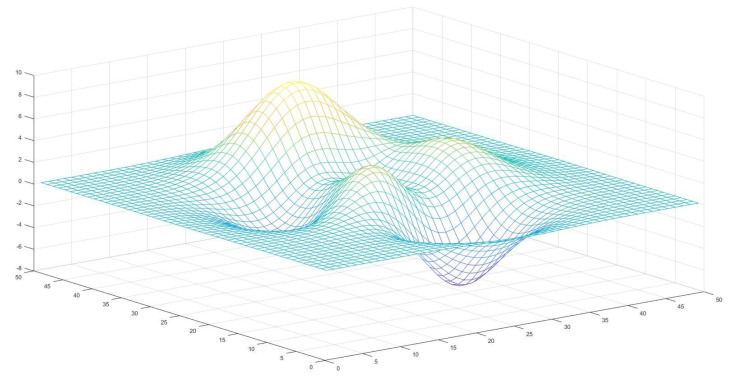
Deployment of 3D topographic maps of sensor nodes.

We used the SAGD algorithm to solve the node deployment problem in 3D sensor networks, using each individual in the SAGD algorithm to represent a combination of sensor node deployments at a time. The 3D terrain is projected downward into a 2D plane, and the first and second dimensions in each individual represent the horizontal and vertical position of the first sensor, the third and fourth dimensions represent the horizontal and vertical position of the second sensor, and so on. When the horizontal and vertical coordinates of the sensors are determined, corresponding to the 3D terrain shown in Figure 10, the height of the sensor nodes is also determined. Now, the whole terrain is divided into one pixel by one, and the signal coverage of the WSN can be expressed using Equation (27).

(27) $$\;\mathrm{rate}\;=\frac{1}{A}\sum_{a=1}^{A}\left(\sum_{b=1}^{B}F\left(S_{b},L_{a}\right)\right)$$

where A denotes the number of pixels over the entire terrain area and B denotes the number of sensor nodes; $F(S_{b},L_{a})$ can be calculated by Equation (26).

To verify the effectiveness of the SAGD algorithm in solving this problem, we compare SAGD with C-PGDRC [51], EBH [52], and MSAALO [53]. All three comparison algorithms were used to solve the 3D sensor node deployment problem. MSAALO is a surrogate-assisted meta-heuristic; the remaining two are improved meta-heuristic algorithms. Experiments for all four algorithms were conducted on the same simulated terrain. To better compare our algorithm with these three algorithms, we set up 30, 40, 50, and 60 sensors for each experiment and ran the experiment 20 times to take the average. The experimental results are shown in Table 7, where N represents the unknown data. The number of real evaluations was 1000 for the SAGD algorithm and MSAALO algorithm and more than 1000 for both the EBH algorithm and the C-PGDRC algorithm.

From Table 7, we can see that as the number of nodes increases, the coverage rate of the WSN also increases. Surrogate-assisted meta-heuristic algorithms have significant advantages over ordinary meta-heuristic algorithms. Our algorithm showed promising results on all node numbers compared to the other three algorithms. The standard deviation was not given in the original paper of the comparison algorithms, so only the standard deviation of the SAGD algorithm is provided here. Through the standard deviation of the SAGD algorithm, we can see that the SAGD algorithm has strong stability when solving such problems, showing its superiority.

## 5. Conclusions

Our method uses some excellent sample information to construct local surrogate models, and we proposed an add-point strategy based on the information from historical surrogate models. The information of each constructed surrogate model was stored in the database. We can select more competitive individuals for real fitness evaluation through the information of historical surrogate models. Two efficient meta-heuristic algorithms, GOA and DE, train the samples. The combination of GOA and DE algorithms enhances the surrogate model’s local exploitation ability and global search ability, and the algorithm can escape from the local optima through the control strategy. A generation-based restart strategy is also incorporated for selecting better samples to avoid GOA and DE being misled by overfitted surrogate models. RBF models are updated every K generations, allowing both meta-heuristic algorithms to be fully explored.

We tested the SAGD algorithm using seven commonly used benchmark functions, and the experimental results show that the SAGD algorithm obtains good results in several dimensions. Finally, the SAGD algorithm was used to solve the deployment problem of 3D sensor nodes. It would be good to apply the SAGD algorithm to more practical problems in future work; the degree of fit of various meta-heuristic algorithms and different surrogate models is also worth considering.

## Figures and Tables

**Table 2:** Comparison of statistical results on 10D.

Function	Method	Mean	Best	Worst	Std.	WS Rank-Test
Ellipsoid	GOA	32.05986	231.7146	139.4887	53.92827	+
	DE	106.5104	225.3724	152.0998	39.62943	+
	GORS-SSLPSO	0.002595	0.113398	0.045255	0.034015	+
	FHSAPPSO	0.7728	8.68944	3.14932	2.43648	+
	SAGD	$4.07\times 10^{-7}$	0.000191	$6.05\times 10^{-5}$	$7.55\times 10^{-5}$	/
Rosenbrock	GOA	93.29924	1163.463	579.0592	378.2185	+
	DE	408.0515	1489.294	847.2024	350.04	+
	GORS-SSLPSO	11.5727	31.6099	20.2086	7.74637	+
	FHSAPPSO	41.0687	82.0577	59.6458	12.6012	+
	SAGD	8.78181	8.88456	8.83557	0.034997	/
Ackley	GOA	16.8616	20.3729	18.8738	1.19643	+
	DE	19.2459	20.6187	19.8791	0.414064	+
	GORS-SSLPSO	3.27872	7.94311	5.13048	1.56844	+
	FHSAPPSO	7.54282	11.3165	9.58837	1.36459	+
	SAGD	0.003649	0.361466	0.190619	0.131978	/
Griewank	GOA	23.3198	130.8887	88.14022	34.36457	+
	DE	36.35064	154.4768	108.8823	33.02842	+
	GORS-SSLPSO	0.292505	1.03913	0.676105	0.281672	+
	FHSAPPSO	1.0836	1.69394	1.32715	0.184562	+
	SAGD	0.002149	0.110366	0.024866	0.033259	/
Rastrigin	GOA	73.54777	134.3728	100.5732	17.1564	+
	DE	85.33661	129.4805	104.5171	14.1654	+
	GORS-SSLPSO	10.0731	76.0396	32.594	19.3827	+
	FHSAPPSO	46.1198	86.8938	74.9257	12.5114	+
	SAGD	$8.45\times 10^{-8}$	0.003516	0.000478	0.001099	/

**Table 3:** Comparison of statistical results on 20D.

Function	Method	Mean	Best	Worst	Std.	WS Rank-Test
Ellipsoid	GOA	260.4076	1073.858	768.2923	212.7306	+
	DE	696.4224	1084.811	865.7986	112.3264	+
	GORS-SSLPSO	0.102103	0.43027	0.247098	0.119181	+
	FHSAPPSO	6.24626	23.0675	14.9304	5.13703	+
	SAGD	$9.50\times 10^{-9}$	$3.21\times 10^{-5}$	$1.17\times 10^{-5}$	$1.16\times 10^{-5}$	/
Rosenbrock	GOA	538.3364	3697.224	2064.123	989.6387	+
	DE	2525.756	4328.362	3342.573	556.2642	+
	GORS-SSLPSO	30.18328	74.89486	48.28018	16.5743	+
	FHSAPPSO	66.58076	116.4849	96.65638	16.72317	+
	SAGD	18.62779	18.76486	18.72371	0.036755	/
Ackley	GOA	13.266	20.394	18.8016	2.5611	+
	DE	19.8995	20.6231	20.362	0.208332	+
	GORS-SSLPSO	1.127967	10.49669	4.345661	2.535612	+
	FHSAPPSO	6.635	9.6644	7.79167	1.01214	+
	SAGD	0.007337	0.106095	0.039336	0.034759	/
Griewank	GOA	170.0912	381.3964	269.2433	60.03781	+
	DE	217.6609	367.7392	315.7809	46.80688	+
	GORS-SSLPSO	0.153632	0.471566	0.354487	0.092308	+
	FHSAPPSO	1.35794	2.17352	1.7729	0.269687	+
	SAGD	$6.05\times 10^{-5}$	0.023478	0.004428	0.007241	/
Rastrigin	GOA	128.1745	277.8843	221.5422	45.55824	+
	DE	238.623	276.5483	257.252	10.93466	+
	GORS-SSLPSO	14.9283	188.9656	47.43567	50.62116	+
	FHSAPPSO	117.9776	183.3136	151.9138	21.55732	+
	SAGD	$1.04\times 10^{-9}$	$5.03\times 10^{-6}$	$6.67\times 10^{-7}$	$1.58\times 10^{-6}$	/

**Table 4:** Comparison of statistical results on 30D.

Function	Method	Mean	Best	Worst	Std.	WS Rank-Test
Ellipsoid	GOA	579.2367	2232.486	1627.833	489.0814	+
	DE	1817.55	2406.832	2064.727	173.2234	+
	GORS-SSLPSO	0.385622	1.571643	0.779207	0.364867	+
	FHSAPPSO	13.79	47.3297	31.8983	12.7156	+
	SAGD	$3.55\times 10^{-9}$	$1.99\times 10^{-5}$	$4.67\times 10^{-6}$	$6.60\times 10^{-6}$	/
Rosenbrock	GOA	837.2743	6979.094	3869.061	1987.883	+
	DE	5140.722	6290.092	5771.844	376.0112	+
	GORS-SSLPSO	63.59973	127.6547	98.75162	20.6104	+
	FHSAPPSO	57.4259	110.7273	84.94207	19.11291	+
	SAGD	28.56271	28.65047	28.59987	0.028426	/
Ackley	GOA	19.1749	20.4097	20.0264	0.36001	+
	DE	20.1485	20.7053	20.5006	0.181577	+
	GORS-SSLPSO	3.425043	7.808269	5.337117	1.479306	+
	FHSAPPSO	6.41898	8.41473	7.44667	0.604243	+
	SAGD	0.00084	0.035894	0.014072	0.014881	/
Griewank	GOA	173.2629	587.0626	365.7254	135.9223	+
	DE	435.1658	613.6942	539.3535	55.08831	+
	GORS-SSLPSO	0.254231	0.535432	0.359967	0.102317	+
	FHSAPPSO	1.98647	3.32629	2.44664	0.470472	+
	SAGD	$5.57\times 10^{-6}$	0.005237	0.001074	0.001853	/
Rastrigin	GOA	281.1905	407.6522	365.2696	37.05603	+
	DE	360.4444	426.2734	401.811	21.96221	+
	GORS-SSLPSO	43.8226	106.875	64.03726	21.26781	+
	FHSAPPSO	187.2826	291.9423	253.7202	26.74184	+
	SAGD	$2.35\times 10^{-11}$	$1.57\times 10^{-6}$	$2.06\times 10^{-7}$	$4.87\times 10^{-7}$	/

**Table 5:** Comparison of statistical results on 50D.

Function	Method	Mean	Best	Worst	Std.	WS Rank-Test
Ellipsoid	GOA	343.7796	4456.966	2216.847	1485.085	+
	DE	6217.867	7707.067	6902.88	528.7414	+
	GORS-SSLPSO	0.002329	471.9035	84.07133	178.5927	+
	FHSAPPSO	5.968347	10.16186	8.153771	1.424026	+
	SAGD	$4.08\times 10^{-12}$	0.000424	$4.99\times 10^{-5}$	0.000133	/
Rosenbrock	GOA	1236.391	7156.267	3824.757	1677.912	+
	DE	9785.587	14020.26	12205.94	1375.941	+
	GORS-SSLPSO	42.27734	95.48175	56.43726	20.29723	=
	FHSAPPSO	49.46244	74.56512	55.93361	8.948982	+
	SAGD	47.10447	48.33749	47.91033	0.453592	/
Ackley	GOA	7.96162	20.5933	14.9695	4.83463	+
	DE	20.4389	20.8575	20.7208	0.123546	+
	GORS-SSLPSO	2.81405	6.351879	4.09504	1.037834	+
	FHSAPPSO	2.934269	4.308483	3.708659	0.445261	+
	SAGD	$7.79\times 10^{-11}$	$3.01\times 10^{-7}$	$3.06\times 10^{-8}$	$9.50\times 10^{-8}$	/
Griewank	GOA	13.56748	815.6762	353.2857	333.8933	+
	DE	776.5821	1066.333	939.6976	87.98558	+
	GORS-SSLPSO	$2.81\times 10^{-5}$	0.017321	0.003338	0.005807	+
	FHSAPPSO	1.065466	1.253401	1.153893	0.062102	+
	SAGD	$1.06\times 10^{-12}$	$8.05\times 10^{-8}$	$8.09\times 10^{-9}$	$2.54\times 10^{-8}$	/
SRR	GOA	699.6644	1088.573	866.0672	121.4055	+
	DE	881.3973	1155.842	1087.913	78.80308	+
	GORS-SSLPSO	−135.396	−24.3956	−73.1469	35.7645	+
	FHSAPPSO	-80.0944	292.118	102.1862	108.9186	+
	SAGD	−212.506	249.6789	−110.814	133.0928	/
RHC	GOA	1315.821	1438.554	1379.788	40.68161	+
	DE	1365.368	1450.478	1421.531	33.20405	+
	GORS-SSLPSO	948.3738	1128.276	1051.301	65.45301	+
	FHSAPPSO	1031.219	1177.053	1080.808	45.1294	+
	SAGD	910	910.0001	910	$3.22\times 10^{-5}$	/

**Table 6:** Comparison of statistical results on 100D.

Function	Method	Mean	Best	Worst	Std.	WS Rank-Test
Ellipsoid	GOA	1435.995	18014.61	8009.537	5426.974	+
	DE	30937.04	34202.67	32093.35	925.4301	+
	GORS-SSLPSO	1.340528	2450.227	514.2376	813.412	+
	FHSAPPSO	64.20482	121.9	85.32166	15.5251	+
	SAGD	$1.39\times 10^{-8}$	0.003632	0.000573	0.001138	/
Rosenbrock	GOA	5404.478	29669.03	15022.01	7685.385	+
	DE	29923.21	33170.58	31613.67	1138.18	+
	GORS-SSLPSO	96.21071	147.2883	102.529	15.74872	−
	FHSAPPSO	130.4009	186.9138	153.22	17.92665	=
	SAGD	97.88537	98.00564	97.96917	0.045568	/
Ackley	GOA	8.32222	20.6833	17.6219	4.57513	+
	DE	20.9042	21.051	20.965	0.049507	+
	GORS-SSLPSO	6.227734	13.78327	10.19949	2.843565	+
	FHSAPPSO	5.12329	8.938998	6.382234	1.180745	+
	SAGD	0.002207	0.019305	0.009207	0.005136	/
Griewank	GOA	91.09843	1946.847	875.4752	677.6542	+
	DE	2156.578	2457.735	2313.977	105.8374	+
	GORS-SSLPSO	0.095989	90.34198	18.22478	37.98808	+
	FHSAPPSO	3.323062	7.230652	4.446424	1.069094	+
	SAGD	$2.51\times 10^{-5}$	0.004696	0.001563	0.001654	/
SRR	GOA	1974.117	2836.632	2346.489	323.2095	+
	DE	2536.289	3086.053	2879.253	180.671	+
	GORS-SSLPSO	1005.055	1266.676	1133.157	94.69411	−
	FHSAPPSO	1106.103	1564.587	1382.903	139.4766	+
	SAGD	1054.448	1697.692	1406.459	209.4309	/
RHC	GOA	1338.608	1581.625	1457.951	81.98479	+
	DE	1470.638	1578.847	1528.803	29.61393	+
	GORS-SSLPSO	1371.871	1482.253	1436.112	32.37645	+
	FHSAPPSO	1282.987	1360.504	1309.62	23.53878	+
	SAGD	910	910.0125	910.0023	0.0043	/

**Table 7:** Comparing results with different numbers of nodes.

Num	EBH	C-PGDRC	MSAALO	SAGD (STD)
30	48.01%	47.55%	47.56%	51.36% (0.0166)
40	57.85%	57.40%	59.45%	62.72% (0.0191)
50	65.06%	64.99%	66.87%	71.36% (0.0179)
60	71.26%	71.27%	N	77.08% (0.0180)

## Data Availability

Not applicable.

## Abbreviations

The following abbreviations are used in this manuscript:

GOA	Gannet optimization algorithm
DE	Differential evolution
SAGD	Combining surrogate-assisted model with GOA and the DE algorithm
WSN	wireless sensor network
RBF	Radial basis function
GP	Gaussian process
LHS	Latin hypercube sampling
GA	Genetic algorithm
PSO	Particle swarm optimization
PPE	Phasmatodea population evolution
WOA	Whale optimization algorithm
SFLA	Shuffled frog leaping algorithm
CR	Crossover probability
LOS	Line of sight
SDB	Sample database
SADB	Surrogate model database
ps	Population size
tpc	An integer tpc in the range of [1, ps]
FESmax	Maximum number of iterations
GORS-SSLPSO	Generation-based optimal restart strategy for surrogate-assisted social learning particle swarm optimization.
FHSAPPSO	Fuzzy hierarchical surrogate assists probabilistic particle swarm optimization
SRR	Shifted rotated rastrigin
RHC	Rotated hybrid composition
SADE	Part of SAGD without the GOA
SAGOA	Part of SAGD without the DE algorithm
C-PGDRC	Parallel gases brownian motion optimization
EBH	Enhanced black hole
MSAALO	Mahalanobis surrogate-assisted ant lion optimization

## References

[1] Jamshed M.A., Ali K., Abbasi Q.H., Imran M.A., Ur-Rehman M. (2022). Challenges, applications and future of wireless sensors in Internet of Things: A review. IEEE Sens. J..

[2] Zhao Q., Li C., Zhu D., Xie C. (2022). Coverage Optimization of Wireless Sensor Networks Using Combinations of PSO and Chaos Optimization. Electronics.

[3] Akay B., Karaboga D. (2012). Artificial bee colony algorithm for large-scale problems and engineering design optimization. J. Intell. Manuf..

[4] Bayzidi H., Talatahari S., Saraee M., Lamarche C.P. (2021). Social network search for solving engineering optimization problems. Comput. Intell. Neurosci..

[5] Cheng M.Y., Prayogo D. (2014). Symbiotic organisms search: A new metaheuristic optimization algorithm. Comput. Struct..

[6] Saremi S., Mirjalili S., Lewis A. (2017). Grasshopper optimisation algorithm: Theory and application. Adv. Eng. Softw..

[7] Moh’d Alia O., Al-Ajouri A. (2016). Maximizing wireless sensor network coverage with minimum cost using harmony search algorithm. IEEE Sens. J..

[8] Wu C., Fu S., Li T. (2017). Research of The WSN Routing based on Artificial Bee Colony Algorithm. J. Inf. Hiding Multim. Signal Process..

[9] Wu C.M., Yang T. (2017). An Improved DV-HOP Algorithm was Applied for The Farmland Wireless Sensor Network. J. Inf. Hiding Multim. Signal Process..

[10] Kaveh A., Seddighian M. (2022). Domain decomposition of finite element models utilizing eight meta-heuristic algorithms: A comparative study. Mech. Based Des. Struct. Mach..

[11] Temel S., Unaldi N., Kaynak O. (2013). On deployment of wireless sensors on 3-D terrains to maximize sensing coverage by utilizing cat swarm optimization with wavelet transform. IEEE Trans. Syst. Man Cybern. Syst..

[12] Jin Y. (2011). Surrogate-assisted evolutionary computation: Recent advances and future challenges. Swarm Evol. Comput..

[13] Luo J., Gupta A., Ong Y.S., Wang Z. (2018). Evolutionary optimization of expensive multiobjective problems with co-sub-Pareto front Gaussian process surrogates. IEEE Trans. Cybern..

[14] Kleijnen J.P. (2009). Kriging metamodeling in simulation: A review. Eur. J. Oper. Res..

[15] Zhou Z., Ong Y.S., Nguyen M.H., Lim D. A study on polynomial regression and Gaussian process global surrogate model in hierarchical surrogate-assisted evolutionary algorithm. Proceedings of the 2005 IEEE Congress on Evolutionary Computation.

[16] Gunn S.R. (1998). Support vector machines for classification and regression. ISIS Tech. Rep..

[17] Billings S.A., Zheng G.L. (1995). Radial basis function network configuration using genetic algorithms. Neural Netw..

[18] Yu H., Tan Y., Sun C., Zeng J., Jin Y. An adaptive model selection strategy for surrogate-assisted particle swarm optimization algorithm. Proceedings of the 2016 IEEE Symposium Series on Computational Intelligence (SSCI).

[19] Elsayed K., Lacor C. (2014). Robust parameter design optimization using Kriging, RBF and RBFNN with gradient-based and evolutionary optimization techniques. Appl. Math. Comput..

[20] Dong H., Li C., Song B., Wang P. (2018). Multi-surrogate-based Differential Evolution with multi-start exploration (MDEME) for computationally expensive optimization. Adv. Eng. Softw..

[21] Díaz-Manríquez A., Toscano G., Coello Coello C.A. (2017). Comparison of metamodeling techniques in evolutionary algorithms. Soft Comput..

[22] Cheng K., Lu Z., Ling C., Zhou S. (2020). Surrogate-assisted global sensitivity analysis: An overview. Struct. Multidiscip. Optim..

[23] Yu H., Tan Y., Sun C., Zeng J. (2019). A generation-based optimal restart strategy for surrogate-assisted social learning particle swarm optimization. Knowl. Based Syst..

[24] Pan J.S., Liu N., Chu S.C., Lai T. (2021). An efficient surrogate-assisted hybrid optimization algorithm for expensive optimization problems. Inf. Sci..

[25] Wang H., Jin Y., Doherty J. (2017). Committee-based active learning for surrogate-assisted particle swarm optimization of expensive problems. IEEE Trans. Cybern..

[26] Stein M. (1987). Large sample properties of simulations using Latin hypercube sampling. Technometrics.

[27] Ma Y., Xiao Y., Wang J., Zhou L. (2021). Multicriteria optimal Latin hypercube design-based surrogate-assisted design optimization for a permanent-magnet vernier machine. IEEE Trans. Magn..

[28] Gunst R.F., Mason R.L. (2009). Fractional factorial design. Wiley Interdiscip. Rev. Comput. Stat..

[29] Won K.S., Ray T. Performance of kriging and cokriging based surrogate models within the unified framework for surrogate assisted optimization. Proceedings of the 2004 Congress on Evolutionary Computation (IEEE Cat. No. 04TH8753).

[30] Jin R., Chen W., Simpson T.W. (2001). Comparative studies of metamodelling techniques under multiple modelling criteria. Struct. Multidiscip. Optim..

[31] Cox D.D., John S. A statistical method for global optimization. Proceedings of the 1992 IEEE International Conference on Systems, Man, and Cybernetics.

[32] Kushner H.J. (1964). A new method of locating the maximum point of an arbitrary multipeak curve in the presence of noise. J. Basic Eng..

[33] Carpio R.R., Giordano R.C., Secchi A.R. (2017). Enhanced surrogate assisted global optimization algorithm based on maximizing probability of improvement. Computer Aided Chemical Engineering.

[34] Forrester A.I., Keane A.J. (2009). Recent advances in surrogate-based optimization. Prog. Aerosp. Sci..

[35] Yu M., Li X., Liang J. (2020). A dynamic surrogate-assisted evolutionary algorithm framework for expensive structural optimization. Struct. Multidiscip. Optim..

[36] Whitley D. (1994). A genetic algorithm tutorial. Stat. Comput..

[37] Loukhaoukha K. (2012). On the security of digital watermarking scheme based on SVD and tiny-GA. J. Inf. Hiding Multimed. Signal Process..

[38] Das S., Mullick S.S., Suganthan P.N. (2016). Recent advances in differential evolution—An updated survey. Swarm Evol. Comput..

[39] Poli R., Kennedy J., Blackwell T. (2007). Particle swarm optimization. Swarm Intell..

[40] Wang H., Wu Z., Rahnamayan S., Liu Y., Ventresca M. (2011). Enhancing particle swarm optimization using generalized opposition-based learning. Inf. Sci..

[41] Sun C., Jin Y., Zeng J., Yu Y. (2015). A two-layer surrogate-assisted particle swarm optimization algorithm. Soft Comput..

[42] Pan J.S., Zhang L.G., Wang R.B., Snášel V., Chu S.C. (2022). Gannet optimization algorithm: A new metaheuristic algorithm for solving engineering optimization problems. Math. Comput. Simul..

[43] Song P.C., Chu S.C., Pan J.S., Yang H. (2022). Simplified Phasmatodea population evolution algorithm for optimization. Complex Intell. Syst..

[44] Zhang L.G., Fan F., Chu S.C., Garg A., Pan J.S. (2021). Hybrid Strategy of Multiple Optimization Algorithms Applied to 3-D Terrain Node Coverage of Wireless Sensor Network. Wirel. Commun. Mob. Comput..

[45] Snasel V., Kong L., Tsai P.W., Pan J.S. (2016). Sink Node Placement Strategies based on Cat Swarm Optimization Algorithm. J. Netw. Intell..

[46] Pan J.S., Kong L., Sung T.W., Tsai P.W., Snášel V. (2018). *α*-Fraction first strategy for hierarchical model in wireless sensor networks. J. Internet Technol..

[47] Nguyen T.T., Lin W.W., Vo Q.S., Shieh C.S. (2021). Delay aware routing based on queuing theory for wireless sensor networks. Data Sci. Patten Recognit..

[48] Xue X., Pan J.S. (2018). A compact co-evolutionary algorithm for sensor ontology meta-matching. Knowl. Inf. Syst..

[49] Chu S.C., Du Z.G., Peng Y.J., Pan J.S. (2021). Fuzzy hierarchical surrogate assists probabilistic particle swarm optimization for expensive high dimensional problem. Knowl. Based Syst..

[50] Suganthan P.N., Hansen N., Liang J.J., Deb K., Chen Y.P., Auger A., Tiwari S. (2005). Problem definitions and evaluation criteria for the CEC 2005 special session on real-parameter optimization. KanGAL Rep..

[51] Gao M., Pan J.S., Li J.P., Zhang Z.P., Chai Q.W. (2021). 3-D terrains deployment of wireless sensors network by utilizing parallel gases brownian motion optimization. J. Internet Technol..

[52] Pan J.S., Chai Q.W., Chu S.C., Wu N. (2020). 3-D terrain node coverage of wireless sensor network using enhanced black hole algorithm. Sensors.

[53] Li Z., Chu S.C., Pan J.S., Hu P., Xue X. (2022). A Mahalanobis Surrogate-Assisted Ant Lion Optimization and Its Application in 3D Coverage of Wireless Sensor Networks. Entropy.

